# Evaluation of Positive Predictors of Micrometastasis in Central Lymph Nodes in Patients with Papillary Thyroid Cancer; A Cross-Sectional Study

**DOI:** 10.30699/IJP.2022.548679.2838

**Published:** 2022-09-02

**Authors:** Hamid Asherloo, Seyed Ali Nabipoorashrafi, Atefeh Kashanizadeh, Mohammad Moradi, Majid Rezaei Tavirani, Massoud Baghai Wadji

**Affiliations:** *Department of General Surgery, School of Medicine, Iran University of Medical Sciences, Tehran, Iran*

**Keywords:** Central lymph nodes, Micrometastasis, Papillary thyroid cancer

## Abstract

**Background & Objective:**

Papillary thyroid cancer (PTC) is the most common primary cancer originating from thyroid follicular cells. The aim of this study was to evaluate the positive predictors of micrometastasis in central lymph nodes in patients with papillary thyroid cancer.

**Methods:**

This was a cross-sectional study. The study population was all known PTC patients who underwent total thyroidectomy and lymph node dissection of the central neck nodes based on the current indications. Confirmation of central lymph node involvement was performed by permanent smear after surgery. Data were analyzed using SPSS software version 22. A P-value below 0.05 was considered statistically significant.

**Results:**

There was no significant relationship between age, gender, family history of PTC, family history of thyroid disease, multinodularity, history of other thyroid diseases, involvement of two thyroid lobes, and tumor grade with central lymph node involvement (*P*>0.05). There was a significant relationship between the tumor pathology and size with central lymph node involvement (*P*<0.05). Moreover, logistic multivariate regression analysis showed that female gender, multinodularity, and tumor size had a significant relationship with the incidence of central lymph node involvement (*P*<0.05).

**Conclusion:**

Female gender, multinodularity, and larger tumor size may be predictors of micrometastasis in central lymph nodes in patients with papillary thyroid cancer.

## Introduction

Papillary thyroid cancer (PTC) is the most common primary cancer originating from thyroid follicular cells. PTC usually has a good prognosis and extended survival for patients (1, 2). This type of cancer is more common in young and middle-aged people and is more prevalent in women (3, 4). 

Papillary carcinoma shows a strong lymphotropism, causing multifocal disease within the thyroid gland, and tends to metastasize to regional lymph nodes (5, 6). The prognosis of papillary thyroid cancer is excellent, with appropriate treatment and a 10-year survival rate of more than 90% (7). 

There is disagreement among surgeons regarding the appropriate treatment following lobectomy and confirmation of PTC in the absence of clinical symptoms (3, 8). Despite this fact, there is almost a general agreement to perform a complete thyroidectomy in high-risk patients (over 45 years, positive lymph nodes, large tumors, and extracapsular invasion.) (9-11). For this reason, some surgeons do not agree with a complete thyroidectomy and believe that treatment of the complications of thyroid reoperation is more difficult than treatment of the tumor itself and suggest radioactive iodine to remove remnant tumors. On the other hand, most surgeons and endocrinologists agree that a complete thyroid is the standard treatment for papillary thyroid cancer (12-14).

Central lymph nodes are a collection of lymph nodes, including the pre-laryngeal, pre-tracheal, and para-tracheal, namely the lymph nodes of level 6 (3, 6). Due to their proximity and role in lymphatic drainage of the thyroid gland, the necessity of their prophylactic dissection in PTC has always been disputed (15-17). One of the clinical gaps is the lack of agreed-upon predictive criteria for clinical decision-making regarding the preservation or removal of central lymph nodes in total thyroidectomy patients with PTC (18, 19). Therefore, the aim of this study was to assess the positive predictors of micrometastasis in central lymph nodes in patients with papillary thyroid cancer who had normal sonography. This study would help surgeons decide which patients might benefit from a lymph node dissection in pre-operation assessment.

## Material and Methods

This was an analytical cross-sectional study. All eligible patients referred to the surgical clinic of Firoozgar Hospital from October 2020 to October 2021 entered the study by available sampling method. Based on the current indications, the study population was all known PTC patients who underwent total thyroidectomy and resection of central neck lymph nodes. PTC was confirmed by needle aspiration pathology (FNA) or based on sonographic TRADS. In some patients, FNA might have been performed despite a hot nodule on the nuclear scan, according to the practitioner's suspicion. After the operation, the pathology study of the permanent smear verified central lymph node involvement. All patients underwent an ultrasound of the neck's central and lateral lymph nodes before surgery.

**Data Analysis**

Data entered SPSS version 22 (SPSS Inc., Chicago, Ill., USA) and analyzed. To define quantitative descriptive findings, mean and standard deviation, and for qualitative findings, frequency and percentage were reported. The Kolmogorov-Smirnov test was used to evaluate the normality of quantitative data. Independent t-test, one-way ANOVA, and Chi-square were used for normal data, and Mann-Whitney, Kruskal-Wallis, and Fisher's exact tests for abnormal data. A P-value below 0.05 was considered statistically significant. 

## Results

This study included 80 patients with known PTC who met the inclusion criteria. The mean ± SD age of patients was 37.88±13.27 years (19-71 years). Sixty one were males and 19 females. Baseline characteristics of the study population are presented in Table 1. Tumor characteristics are presented in Table 2.

**Table 1:** Baseline characteristics of the study population

Characteristics	With central lymph node involvement N=49	Without central lymph node involvement N=31	P-Value
Age (year)	36.77±14.91	39.65±10.65	0.063
Gender Female n (%) Male n (%)	34 15	27 4	0.105
PTC History	3	0	0.279
Thyroid disease family history	2	1	0.90

**Table 2:** Tumor histological characteristics

Tumor histology	With central lymph node involvement	Without central lymph node involvement	P-Value
Tumor Grade			
1	7	1	0.924
2	1	1	
3	16	11	
4	25	16	
Tumor size (cm)	3.19±2.28	1.2±0.61	<0.001
Both thyroid lobe involvement	47	28	0.37
Multinodularity	11	9	0.599
Tumor Pathology			
Micropapillary thyroid carcinoma	0	3	<0.001
Multi-nodular goiter with adenomatous hyperplasia/PTC	0	3	
Papillary thyroid carcinoma	12	0	
Papillary thyroid carcinoma	37	22	
Nodular thyroid/right lobe/FNA cytology	0	3	

**Fig. 1:**
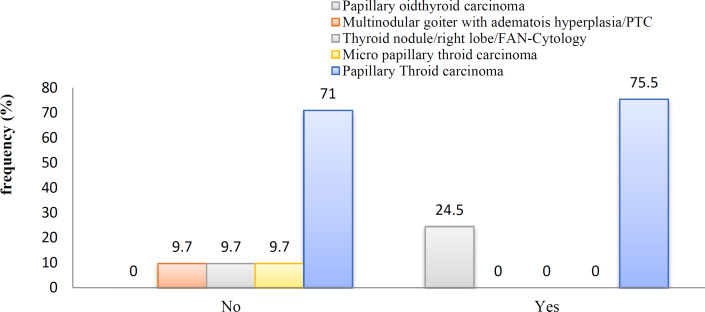
The frequency of tumor pathology in patients with and without central lymph nodes involvement

There was no significant relationship between age, gender, family history of PTC or other thyroidal diseases, tumor grade, multinodularity, and right or left lobe PTC, or both sides, with central lymph node involvement (*P*>0.05). Despite the fact, there was a significant relationship between the tumor pathology and central lymph node involvement (*P*<0.05). Figure 1 shows the frequency of tumor pathology in patients with and without central lymph node involvement.

Moreover, there was a significant relationship between tumor size and central lymph node involvement (*P*<0.05).

Finally, multivariate logistic regression was used to determine factors affecting the involvement of central node lymph nodes. Gender, multinodularity, and tumor size significantly affected central lymph node involvement (*P*<0.05). Multivariate logistic regression analysis of predictors of central lymph node involvement in patients under study is presented in Table 3.

**Table 3:** Multivariate logistic regression analysis of the predictors of central lymph node involvement

Variable	Coefficient	S.E	P-value	Odds ratio
Age	-0.31	0.033	0.339	0.969
Gender	-2.33	1	0.02	0.096
Tumor Pathology	0.049	0.34	0.887	1.049
PTC family history	20.92	22941	0.999	1225544894
Multinodularity	-2.81	1.34	0.036	0.06
Thyroid disease family history	-1.84	1.1	0.094	0.158
Both lobe involvement	-3.05	1.89	0.107	0.047
Tumor size	2.28	0.66	0.001	9.86
Tumor grade	0.005	0.34	0.988	1.006
The distance between the tumor and the nearest nodule	-0.858	0.49	0.081	4.23

## Discussion

Based on the most important results of this study, there was a significant correlation between tumor pathology and size with central lymph node involvement in patients with PTC. Moreover, multivariate logistic regression showed that gender, multinodularity, and tumor size significantly affected the incidence of central lymph node involvement. Therefore, it seems that the female gender, multinodularity, and larger tumor size are predictors of micrometastasis in central lymph nodes in patients with papillary thyroid cancer.

Previous studies have investigated some predictors of positive micrometastasis in central lymph nodes in patients with papillary thyroid cancer (20-27). In the study of Seong* et al.*, in line with the present study, they found that a distance of less than 1.9 mm was a significant correlation as a predictor of central lymph node metastasis. However, no relationship was observed between age, gender, and tumor size (20). Moreover, Zhou* et al.* found that males aged less than 50 years and tumor size larger than 7 mm were independent predictors of central lymph metastases. However, multifocal tumors and bilateral thyroid involvement did not have a significant effect (21). In the study of Kim* et al.*, independent risk factors were tumor size larger than 5 mm, bilateral involvement, extracapsular invasion, and lateral node lymph node metastasis. The highest odds ratio for cervical lymph node metastasis was 15.89 (22).

Furthermore, Howell* et al.* indicated the role of BRAF gene V600E mutation in central lymph node involvement as an independent risk factor. However, age, sex, and tumor size had no independent effect in predicting central node lymph node metastasis. The positive predictive value of BRAF gene mutation was 50% when the frequency of lymph node metastasis was 37% (23).

Furthermore, Mazzaferri* et al.* summarized the positive and negative features of cervical dissection in a commentary. They believed that prophylactic dissection should not be performed routinely due to following complications. Confirmed side effects include transient recurrent nerve damage and transient hypothyroidism (28). They highlighted the importance of risk factor assessment to select patients more appropriately for prophylactic neck dissection or iodine therapy. 

Moghimi* et al.* studied the risk factors for microscopic involvement of the central lymph nodes in patients with papillary thyroid cancer without obvious involvement of the cervical lymph nodes. The incidence of metastasis was about 49%. There was a weak positive correlation between patient age and metastasis and a very weak correlation between tumor size and metastasis. The cut-off point for age was 42.5 years (sensitivity 63% and specificity 62%), and for tumor size was 1.75 cm (sensitivity 58% and specificity 57%). The incidence of metastasis was higher in men than women and in the case of multifocal tumors than single focal ones. The incidence of metastasis was weakly correlated with the tumor's location and the invasion of the thyroid capsule. They concluded that old age, larger tumor size, masculinity, multifocal tumor, and tumor invasion into the capsule were risk factors for central lymph node metastasis, and it may be possible to perform prophylactic lymph node dissection in such patients (29). Their findings were inconsistent with our study in many aspects, possibly due to the small sample size or study design in the two investigations. 

In addition, Choi* et al.* evaluated cervical lymph node metastasis in papillary thyroid carcinoma in 589 patients who underwent surgery due to PTC. An increased risk of lymph node metastasis was observed in male patients less than 45 years, tumor size> 1 cm, vascular, lymphatic invasion, and extrathyroidal invasion. Cancers in the upper neck had a relatively higher risk of lateral metastasis than those in the lower parts. Ultrasound (US) and Computed Tomography (CT) specificity for central and lateral lymph node metastasis (LNM) were high (92-97%). Using a central lymph node size greater than 5 mm to indicate metastasis, the US had a preoperative sensitivity of 58.3% and a specificity of 71.4%. US and CT preoperative imaging were suggested to be useful for identifying features that indicate a high-risk LNM and for determining appropriate PTC management (30). Some features of this study were in line with our results, such as the more probability of metastasis in larger tumor sizes, but considering gender, it was not consistent. Such differences are likely to be due to different ethnicities. 

Our study had some limitations. The sample size was quite small, and the follow-up period was short. Few similar studies have been performed; therefore, comparing the present study with other studies is limited. Hence, it is suggested to perform larger multicentric studies to elucidate the risk factors more clearly for central lymph node metastasis. Moreover, writing a systematic review or meta-analysis on this issue is suggested

## Conclusion

Female gender, multinodularity, and larger tumor size may be predictors of micrometastasis in central lymph nodes in patients with papillary thyroid cancer. 

## Ethical Consideration

 No extra expenses were imposed on patients. No change in routine treatment processes was made for any patient for a research purpose. Data were extracted from hospital documents, and therefore no consent was obtained. However, data was used anonymously. The study protocol was approved by the ethics committee of Iran University of Medical Sciences.

## Funding

This was part of a thesis to obtain a specialty degree in general surgery from IUMS by one of the authors. 

## Authors' Contribution

All authors contributed equally to preparing the manuscript. 

## Conflict of Interest

The authors declared no conflict of interest.
